# Supplementary material for: A Higher Polygenic Risk Score Is Associated with a Higher Recurrence Rate of Atrial Fibrillation in Direct Current Cardioversion-Treated Patients
Source: Medicina (Kaunas). 2021 Nov 18;57(11):1263. doi: 10.3390/medicina57111263 (PMC8624440; doi:10.3390/medicina57111263)
Supplement: Supplementary file 1 [file medicina-57-01263-s001.zip › medicina-1415650-supplementary.pdf]

Supplementary Materials:

Figure S1: Description of the study group

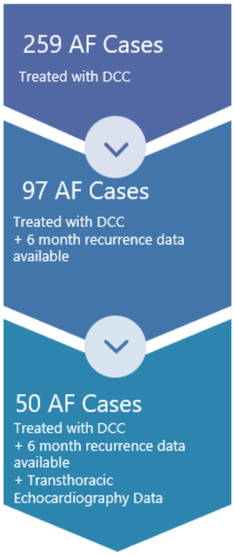

Table S1: Multivariate logistic regression analysis of AF occurrence vs. SNVs

Variables in the Equation									
		B	S.E.	Wald	df	Sig.	Exp(B)	95% C.I.for EXP(B)	
								Lower	Upper
Step 1[a]	CAV1_Risk Allele/Other	-0.190	0.397	0.230	1	0.632	0.827	0.380	1.800
	MYH7_Risk Allele/Other	-0.173	0.341	0.259	1	0.611	0.841	0.431	1.640
	SOX5_Risk Allele/Other	-0.173	0.357	0.235	1	0.628	0.841	0.418	1.694
	ZFHX3_Risk Allele/Other	0.253	0.343	0.545	1	0.460	1.288	0.658	2.520
	KCNN3_Risk Allele/Other	0.247	0.319	0.599	1	0.439	1.280	0.685	2.393
	KCNJ5_Risk Allele/Other	-0.364	0.417	0.759	1	0.384	0.695	0.307	1.575
	PITX2rs6838973_Risk Allele/Other	0.169	0.394	0.184	1	0.668	1.184	0.547	2.563
	PITX2rs2200733_Risk Allele/Other	0.837	0.331	6.382	1	0.012	2.310	1.206	4.423
	Sex	-1.059	0.319	11.024	1	0.001	0.347	0.186	0.648
	Age	0.040	0.017	5.486	1	0.019	1.041	1.007	1.076
	Pulmonary arterial hypertension_combined	0.165	0.394	0.177	1	0.674	1.180	0.545	2.552
	Congestive Heart Failure_combined	2.622	0.405	41.918	1	0.000	13.767	6.224	30.450
	Coronary heart disease	1.765	0.717	6.062	1	0.014	5.844	1.433	23.825
	Stroke	-1.167	0.711	2.699	1	0.100	0.311	0.077	1.253
	Diabetes_Combined	-2.057	0.535	14.801	1	0.000	0.128	0.045	0.365

	Dyslipidemia	-1.551	0.338	21.057	1	0.000	0.212	0.109	0.411
	Chronic Respiratory Disorders	-0.379	0.754	0.253	1	0.615	0.685	0.156	2.999
	BMI	0.085	0.032	7.124	1	0.008	1.089	1.023	1.159
	Constant	-4.080	1.469	7.713	1	0.005	0.017		

a. Variable(s) entered on step 1: Sex, Age, Pulmonary arterial hypertension_combined, Congestive Heart Failure_combined, Coronary heart disease, Stroke, Diabetes_Combined, Dyslipidemia , Chronic Respiratory Disorders, BMI.

Table S2: Multivariate logistic regression analysis of AF occurrence vs. PRS>7

Variables in the Equation

		B	S.E.	Wald	df	Sig.	Exp(B)	95% C.I.for EXP(B)	
								Lower	Upper
Step 1[a]	PRS>;7	-0.195	0.903	0.047	1	0.829	0.823	0.140	4.826
	Sex	-1.156	0.309	13.943	1	0.000	0.315	0.172	0.578
	Age	0.036	0.016	4.979	1	0.026	1.037	1.004	1.071
	Pulmonary arterial hypertension_combined	0.008	0.374	0.000	1	0.983	1.008	0.485	2.097
	Congestive Heart Failure_combined	2.589	0.396	42.764	1	0.000	13.316	6.129	28.932
	Coronary heart disease	1.622	0.703	5.316	1	0.021	5.063	1.275	20.098
	Stroke	-0.960	0.703	1.865	1	0.172	0.383	0.096	1.519
	Diabetes_Combined	-1.959	0.505	15.047	1	0.000	0.141	0.052	0.379
	Dyslipidemia	-1.414	0.319	19.639	1	0.000	0.243	0.130	0.454
	Chronic Respiratory Disorders	-0.322	0.712	0.204	1	0.652	0.725	0.179	2.929
	BMI	0.087	0.031	7.863	1	0.005	1.091	1.027	1.159
	Constant	-3.495	1.374	6.469	1	0.011	0.030		

a. Variable(s) entered on step 1: Sex, Age, Pulmonary arterial hypertension_combined, Congestive Heart Failure_combined, Coronary heart disease, Stroke, Diabetes_Combined, Dyslipidemia , Chronic Respiratory Disorders, BMI.

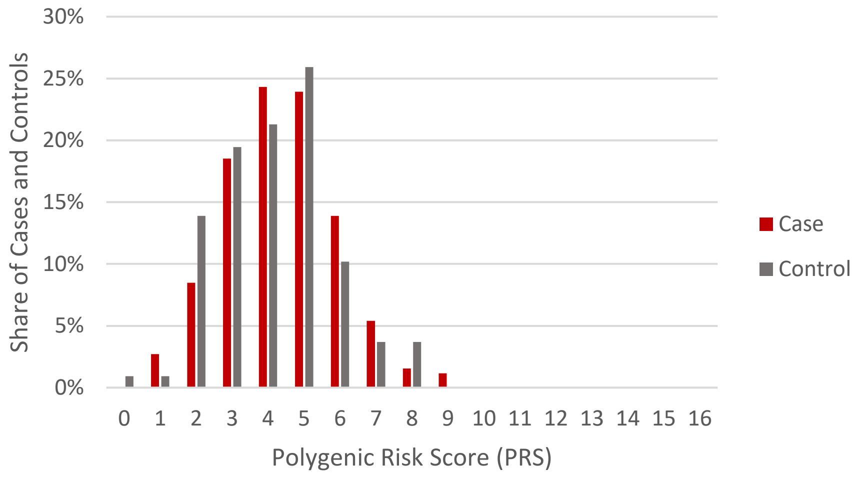

Figure S2. Polygenic Risk Score (PRS) in cases (n=259) and control (n=108) groups. The PRS was calculated as the total unweighted number of risk alleles that a case or control had.

Table S3: Multivariate regression analysis of AF recurrence vs. risk alleles

Variables in the Equation									
		B	S.E.	Wald	df	Sig.	Exp(B)	95% C.I.for EXP(B)	
								Lower	Upper
Step 1[a]	CAV1_Risk Allele/Other	-0.632	0.688	0.844	1	0.358	0.532	0.138	2.046
	MYH7_Risk Allele/Other	0.361	0.575	0.395	1	0.530	1.435	0.465	4.426
	SOX5_Risk Allele/Other	-1.501	0.611	6.033	1	0.014	0.223	0.067	0.738
	ZFHX3_Risk Allele/Other	-0.951	0.535	3.161	1	0.075	0.386	0.136	1.102
	KCNN3_Risk Allele/Other	0.710	0.517	1.889	1	0.169	2.034	0.739	5.598
	KCNJ5_Risk Allele/Other	0.179	0.703	0.065	1	0.799	1.196	0.302	4.741
	PITX2rs6838973_Risk Allele/Other	1.391	0.765	3.308	1	0.069	4.018	0.898	17.978
	PITX2rs2200733_Risk Allele/Other	0.596	0.531	1.258	1	0.262	1.814	0.641	5.137
	CHA2DS2-VASc>1_(Women)_or_>0_(Men)	-0.254	1.120	0.051	1	0.821	0.776	0.086	6.969
	Non_CHA2DS2_Vasc_Comorbidities	-0.504	0.928	0.295	1	0.587	0.604	0.098	3.725
	BMI	-0.014	0.046	0.095	1	0.758	0.986	0.901	1.079
	Duration since initial diagnosis, months	0.015	0.006	5.987	1	0.014	1.015	1.003	1.028
	Age of initial Diagnosis, years	0.044	0.027	2.770	1	0.096	1.045	0.992	1.101
	Constant	-2.393	2.339	1.047	1	0.306	0.091		

a. Variable(s) entered on step 1: BMI, Duration since initial diagnosis, months, Age of initial Diagnosis, years.

Table S4: Multivariate regression analysis of AF recurrence vs. PRS>7

Variables in the Equation									
		B	S.E.	Wald	df	Sig.	Exp(B)	95% C.I.for EXP(B)	
								Lower	Upper
Step 1[a]	PRS>7	1.429	0.538	7.056	1	0.008	4.174	1.454	11.980
	CHA2DS2-VASc>1_(Women)_or_>0_(Men)	-0.567	1.098	0.267	1	0.605	0.567	0.066	4.875
	Non_CHA2DS2_Vasc_Comorbidities	-0.614	0.881	0.485	1	0.486	0.541	0.096	3.046
	BMI	-0.012	0.041	0.088	1	0.766	0.988	0.911	1.071
	Duration since initial diagnosis, months	0.014	0.006	4.979	1	0.026	1.014	1.002	1.026
	Age of initial Diagnosis, years	0.031	0.025	1.535	1	0.215	1.032	0.982	1.084
	Constant	-1.019	1.969	0.268	1	0.605	0.361		

a. Variable(s) entered on step 1: BMI, Duration since initial diagnosis, months, Age of initial Diagnosis, years.

Table S5: Multiple regression analysis including PRS > 7 on AF cases with Transthoracic Echocardiography data (n=50)

Variables in the Equation									
		B	S.E.	Wald	df	Sig.	Exp(B)	95% C.I.for EXP(B)	
								Lower	Upper
Step 1[a]	PRS>7	3.658	1.491	6.015	1	0.014	38.766	2.085	720.952
	Age of initial Diagnosis, years	0.041	0.051	0.655	1	0.418	1.042	0.943	1.151
	Duration since initial diagnosis, months	0.013	0.009	1.970	1	0.160	1.013	0.995	1.031
	Sex	2.921	1.180	6.132	1	0.013	18.569	1.839	187.492
	BMI	-0.152	0.082	3.456	1	0.063	0.859	0.732	1.008
	Comorbidities	-20.948	15715.750	0.000	1	0.999	0.000	0.000	
	EF,%	0.011	0.043	0.071	1	0.791	1.011	0.930	1.099
	LAVI,ml/m2	0.100	0.056	3.171	1	0.075	1.105	0.990	1.233
	Constant	16.921	15715.751	0.000	1	0.999	22313190.144		

a. Variable(s) entered on step 1: Age of initial Diagnosis, years, Duration since initial diagnosis, months, Sex, BMI, Comorbidities, EF,%, LAVI,ml/m2.
